# Application of Millifluidics to Encapsulate and Support Viable Human Mesenchymal Stem Cells in a Polysaccharide Hydrogel

**DOI:** 10.3390/ijms19071952

**Published:** 2018-07-03

**Authors:** Fabien Nativel, Denis Renard, Fahd Hached, Pierre-Gabriel Pinta, Cyril D’Arros, Pierre Weiss, Catherine Le Visage, Jérôme Guicheux, Aurélie Billon-Chabaud, Gael Grimandi

**Affiliations:** 1 Inserm, UMR 1229, RMeS, Regenerative Medicine and Skeleton, Université de Nantes, ONIRIS, F-44042 Nantes, France; fabien.nativel90@gmail.com (F.N.); fahd.hached@univ-nantes.fr (F.H.); Pierre.Pinta@chu-nantes.fr (P.-G.P.); cyril.d-arros@inserm.fr (C.D.); Pierre.Weiss@univ-nantes.fr (P.W.); catherine.levisage@inserm.fr (C.L.V.); jerome.guicheux@univ-nantes.fr (J.G.); Aurelie.Billon@univ-nantes.fr (A.B.-C.); gael.grimandi@univ-nantes.fr (G.G.); 2 CHU Nantes, Pharmacie Centrale, PHU 11, F-44093 Nantes, France; 3 INRA UR1268, Biopolymères Interactions Assemblages, F-44300 Nantes, France; 4 UFR Sciences Pharmaceutiques et Biologiques, Université de Nantes, F-44035 Nantes, France; 5 CHU Nantes, PHU4 OTONN, F-44093 Nantes, France; 6 UFR Odontologie, Université de Nantes, F-44042 Nantes, France

**Keywords:** droplet millifluidics, encapsulation, human adipose-derived stromal cells, hydrogel, self-hardening, silanized-hydroxypropylmethylcellulose, biomedical, degenerative disease

## Abstract

Human adipose-derived stromal cells (hASCs) are widely known for their immunomodulatory and anti-inflammatory properties. This study proposes a method to protect cells during and after their injection by encapsulation in a hydrogel using a droplet millifluidics technique. A biocompatible, self-hardening biomaterial composed of silanized-hydroxypropylmethylcellulose (Si-HPMC) hydrogel was used and dispersed in an oil continuous phase. Spherical particles with a mean diameter of 200 μm could be obtained in a reproducible manner. The viability of the encapsulated hASCs in the Si-HPMC particles was 70% after 14 days in vitro, confirming that the Si-HPMC particles supported the diffusion of nutrients, vitamins, and glucose essential for survival of the encapsulated hASCs. The combination of droplet millifluidics and biomaterials is therefore a very promising method for the development of new cellular microenvironments, with the potential for applications in biomedical engineering.

## 1. Introduction

Mesenchymal stromal cells (MSCs) are of significant medical interest as they have the ability to differentiate into several cell types (including chondrocytes, osteocytes, and adipocytes). They have already been exploited to treat several pathologies, including osteo-articular diseases, diabetes, cancer, cardiovascular pathologies, angiogenic diseases, and skin injuries [1,2,3,4,5,6]. In recent years, they have become known for their potent immunomodulatory and anti-inflammatory activities, which stem from their ability to secrete bioactive trophic factors and to release extracellular vesicles [7,8,9]. MSCs can be isolated from a broad range of adult tissues, including bone marrow, adipose tissue, and articular synovial fluid [10].

The literature to date indicates that MSCs injection is subject to two main limitations: extensive cell death due to the mechanical forces during injection, thereby making it difficult to detect the injected cells over a sustained period of time [11,12], and the risk of cell leakage after their injection due to the propensity of MSCs to migrate [13,14]. The encapsulation of MSCs in biomaterials prior to their injection appears to be an alternative strategy for their administration that overcomes these limitations and that facilitates the delivery of therapeutic factors in several pathologies [15,16]. The optimal injection of encapsulated MSCs requires spherical devices with a size that is compatible with a standard needle characteristic.

Cell encapsulation consists in entrapping viable and functional cells within a matrix in order to enable immune isolation by the creation of a physical barrier between the host immune system and the transplanted tissue [17]. Moreover, cell encapsulation enhances the retention of the cells at the targeted tissue and protects them from the mechanical damage that occurs with injection. Indeed, the viability of the cells is compromised as they are subjected first to shear stress during syringe needle flow and secondly to stretching forces and deformations due to extensional flow during syringe needle ejection [12]. The matrix must be biocompatible and semi-permeable to obtain the intended bio-functionality. Indeed, it must not hinder the diffusion of essential factors required for cell survival (e.g., oxygen and nutrients derived from the blood and waste products of cellular metabolism). Most of the biomaterials used for cell encapsulation are hydrogels, in which the cells are embedded in a fully hydrated matrix. This allows for the survival of the encapsulated cells and their therapeutic actions. Cell encapsulation is carried out by mixing the cells with polymeric solutions that are then crosslinked under operating conditions that depend on the nature of the polymer. By protecting the transplanted tissue, encapsulation can improve the safety of cell therapies [17]. This approach was first proposed by Lim et al. in 1980, who encapsulated pancreatic cells in alginate prior to their injection into diabetic rats so as to be able to avoid immunosuppressive therapy [18].

Alginate is the most extensively investigated and characterized polymer for cell encapsulation due to its intrinsic properties, such as biocompatibility, and the requirements of calcium ions for crosslinking [19]. Other natural polymers such as agarose may support viable cells by constituting microenvironments that mimic natural tissues [20]. However, alginate hydrogels are sensitive to non-gelling agents (such as sodium ions) in physiological solutions. Indeed, a sodium-calcium exchange occurs under physiological conditions, leading to the disruption of the hydrogel [21]. To overcome these limitations, our laboratory favors the use of an injectable, biocompatible, self-hardening hydrogel composed of silanized-hydroxypropylmethylcellulose (Si-HPMC). This labeled hydrogel has been reported to support both cell viability and bio-functionality after encapsulation [22,23,24]. In 2008, in vivo evaluations after implantation of a ruthenium-labeled Si-HPMC hydrogel into rabbit bone defects showed that this polymer is biocompatible and not degraded until at least the eighth week [25].

Several techniques are currently used for cell encapsulation [26,27]. For example, emulsions can be used to encapsulate cells within spherical structures referred to as “particles” [28]. Recently, Hached et al. [29] encapsulated cells in Si-HPMC particles using a water-in-oil dispersion protocol. The MSCs were found to have long-term viability and the ability to secrete immunomodulatory and anti-inflammatory factors [29]. However, this method has not been able to reproducibly generate Si-HPMC particles with a monodispersed size that are optimal for in vivo studies. In contrast to dispersion/emulsification techniques, microfluidics technology allows the highly reproducible generation of uniform microparticles with a controlled size [30,31]. Microfluidics has generated significant interest in the research of cell encapsulation [32]. Another approach, called millifluidics, which bears similarities to microfluidics, has ample potential for use in cell encapsulation.

Millifluidics is characterized by the assembly of inexpensive and commercially available millimeter-sized tubing and chromatography connectors. Aside from the need for syringe pumps, this method is easy to implement and does not require specific equipment. The emergence of millifluidics is a relatively new phenomenon. The first millifluidic device, which was referred to as a “simplified microfluidic device”, was developed by Quevedo et al. in 2005 [33]. It offers a high level of versatility and rapid creation of a modular setup. It is an efficient tool to investigate polymerization reactions or to create microparticles with controlled and fine-tuned sizes and shapes [34,35,36,37]. Moreover, Amine et al. recently showed that droplet-based millifluidics represents an efficient means to probe the liquid-liquid phase separation of various biopolymers mixtures [38]. Fluidic technologies consist of the introduction of the polymeric solution to be dispersed through a capillary or a needle into the co-flowing continuous phase to generate droplets [39]. The structure of the particles can be determined by the flow properties in the devices while the chemical composition is dictated by the selected fluids. With the use of a basic junction such as a co-axial or T-junction, millifluidics offers more advantages than soft lithography and microfluidic techniques. The millifluidic apparatus is an assembly of capillaries or flexible tubes (plastic or silica tubing with diameters ranging from 50 μm to a few millimeters) connected by elementary home-made or commercial (Upchurch^®^) modules. Commercially available tubes have a range of wettability and transparency properties and they generally exhibit good chemical resistance to pressure and temperature [40]. The elementary modules are able to achieve the basic functions used in microfluidic devices, such as the formation of periodic trains of monodisperse droplets with very good control over their size or the dilution-concentration of these trains while keeping the volume of the droplets unchanged [41]. Modular millifluidic setups can then be designed to produce newly controlled integrated configurations, limited only by the number of combinations possible and one’s creativity [41]. The connecting capillary tubes and the various modules can readily be assembled and disassembled so that modular setups can be designed as needed in a short period of time. The great versatility of this method provided the millifluidics strategy several advantages over microfluidic synthesis, while retaining its suitability for the in-depth study of critical parameters involved in microparticle production. Millifluidics is therefore an inexpensive and versatile method that allows for the production of particles with optimal injection properties for in vivo studies: reproducibility and size monodispersity. The literature indicates that following the optimization of experimental conditions, the particles produced by millifluidics generally have a polydispersity below 2–3% [42].

In this context, millifluidics appears to be a promising approach to control the granulometry of encapsulated MSCs in polysaccharide hydrogel droplets and to prevent the polydispersity of the particles observed in previous studies [29]. This approach provides better control of the numbers of injectable cells and optimizes their viability. The objectives of this study were (i) to verify that the rheological properties of the Si-HPMC hydrogel are consistent with millifluidic techniques, (ii) to characterize the Si-HPMC particles generated, and (iii) to assess the in vitro viability and proliferation of MSCs after their encapsulation.

## 2. Results

### 2.1. Rheological Assessments of the Si-HPMC Solution and Gel

**Figure 1:**
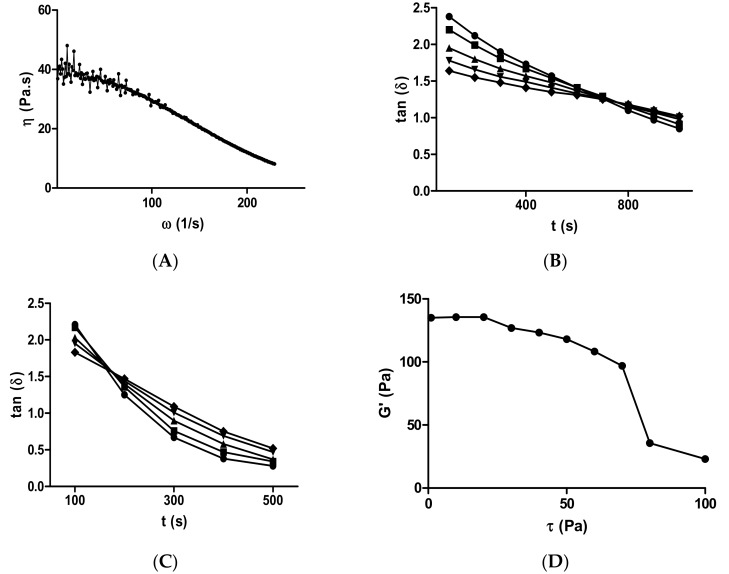
Rheological characterization of silanized-hydroxypropylmethylcellulose (Si-HPMC) solution and gel. (**A**) Flow curves (viscosity vs. shear rate) of a 4% Si-HPMC solution (pH 7.0). (**B**) Tan (δ) vs. time (in order to determine *t_gel_*) of a 2% Si-HPMC hydrogel (pH 7.0) at 23 °C and (**C**) at 37 °C. Tan (δ) was determined at five oscillation frequencies: 0.30 Hz (●), 0.50 Hz (■), 1 Hz (▲), 1.80 Hz (▼), and 3.2 Hz (♦). (**D**) The equilibrium storage modulus (*G*′) was determined for applied stress amplitudes (τ) ranging from 0.1 to 1000 Pa and a fixed frequency of 1 Hz after 24 h for a 2% Si-HPMC hydrogel (pH 7.0) at 37 °C. Each rheological test was repeated three times.

The flow behavior of the Si-HPMC solution showed that the steady shear viscosity decreased as the shear rate increased (Figure 1A). A Newtonian plateau observed at low shear rates was followed by a shear-thinning behavior. At a high shear rate, the viscosity exhibited power-law dependence with the shear rate. The flow curve could readily be fitted to the simplified Cross model using Equation (2) (correlation coefficient of 0.989). The limiting Newtonian viscosity (η_0_) was 38.6 ± 1.2 Pa.s for a 4% (*w*/*w*) non-sterile Si-HPMC.

Instantaneously, after the initiation of Si-HPMC crosslinking upon pH neutralization by the mixing of one volume of HEPES buffer (pH 3.6) with one volume of the Si-HPMC polymer solution, the influence of temperature on the gelation time *t_gel_* was monitored using dynamic frequency measurements (Figure 1B,C). According to the criterion of Winter and Chambon, the cross-over of tan δ with time at the five applied frequencies yielded a gelation time *t_gel_* of 701 ± 29.7 s at 23 °C (Figure 1B) and of 123.3 ± 15.3 s at 37 °C (Figure 1C) for the 2% (*w*/*w*) Si-HPMC hydrogel [43,44].

To further characterize the Si-HPMC hydrogel, the average mesh size, corresponding to the average distance between entanglements in the hydrogel network, was evaluated using Equation (3). The equilibrium storage modulus *G*′ had a value of 124.7 ± 17.9 Pa for 2% Si-HPMC gel at pH 7 (Figure 1D). From this value, an average mesh size (ξ) of 32.1 ± 1.6 nm was calculated.

### 2.2. Characterization of the Shape and the Size of the Si-HPMC Particles

**Figure 2:**
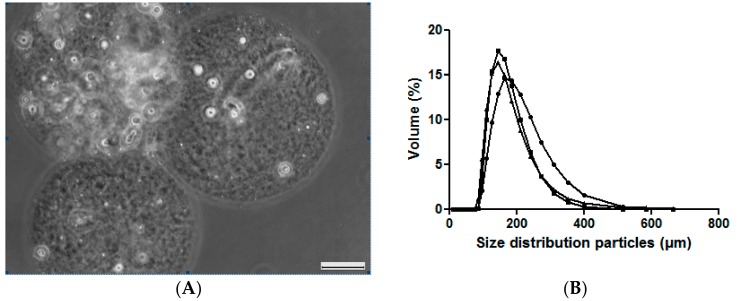
Characterization of the Si-HPMC particles. (**A**) Representative light microscopy image of the Si-HPMC particles produced using droplet-based millifluidics; (**B**) Size distribution of three batches of Si-HPMC particles produced using droplets-based millifluidics and as determined by laser diffraction. Scale bar: 50 μm.

The droplet-based millifluidics process was optimized by the application of a dispersed flow rate of 16 μL/min and a continuous flow rate of 150 μL/min. Under these operating conditions, after the washing and collection of the Si-HPMC particles in phosphate buffered salt (PBS), light microscopy observations revealed that the Si-HPMC particles were spherical and uniform with smooth surfaces and a size of 200 μm (Figure 2A). Laser-based particle size analyses revealed a monomodal population of particles that were between 170 and 210 μm in size, with an average diameter of 192 ± 16 μm (Figure 2B).

### 2.3. Diffusion Properties of the Si-HPMC Particles

Following the incubation of the Si-HPMC particles (with an average diameter of 192 ± 16 μm) in 1 mg/mL solutions of fluorescein isothiocyanate (FITC)-dextran of different molecular weights for 18 h, the diffusion properties of Si-HPMC hydrogels were assessed using confocal laser scanning microscopy (CLSM).

**Figure 3:**
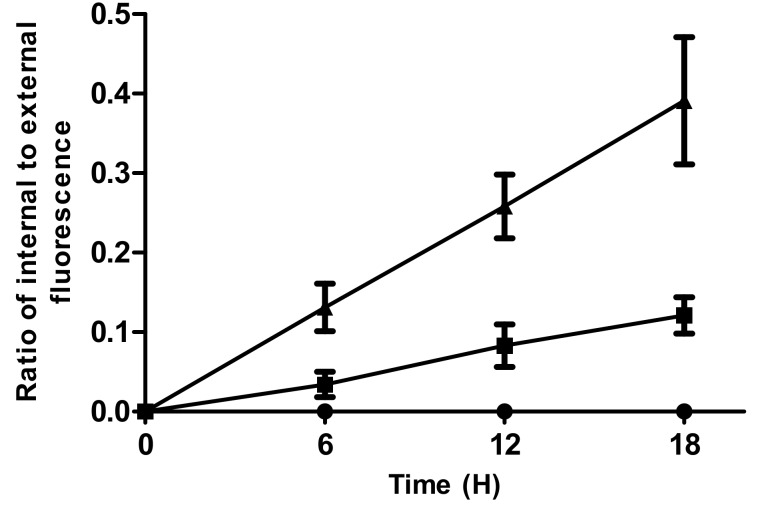
Diffusion properties of the Si-HPMC particles. Particles of Si-HPMC were incubated with FITC-dextran (M_w_ 20 kDa (▲), 250 kDa (■), and 2000 kDa (●)) solutions for 18 h. The ratio of the maximum fluorescence intensity inside and outside the particles was calculated, after assessment of the fluorescence intensities of the particles (inside) and the FITC-dextran solutions (outside) using confocal laser scanning microscopy (CLSM). Si-HPMC particles with 192 ± 16 μm diameters were selected for this study. Each test was performed for one particle at a time and repeated three times.

At the beginning of the experiment, the Si-HPMC particles did not exhibit any fluorescence, which is in agreement with the absence of fluorescence signals emanating from the Si-HPMC polymer. An increase in fluorescence intensity was then noticed as a function of time for the Si-HPMC particles incubated with 20 kDa and 250 kDa FITC-dextrans (Figure 3). After 18 h, the internal to external ratio was 0.39 and 0.1 for the 20 kDa and the 250 kDa fluorescently labeled dextrans, respectively. In addition, no fluorescence was detected inside the Si-HPMC particles after 18 h for the fluorescently labeled 2000 kDa dextran.

### 2.4. Evaluation of Encapsulated Human Adipose-Derived Stromal Cells (hASCs) Viability and Estimation of the Average Number of Encapsulated hASCs

**Figure 4:**
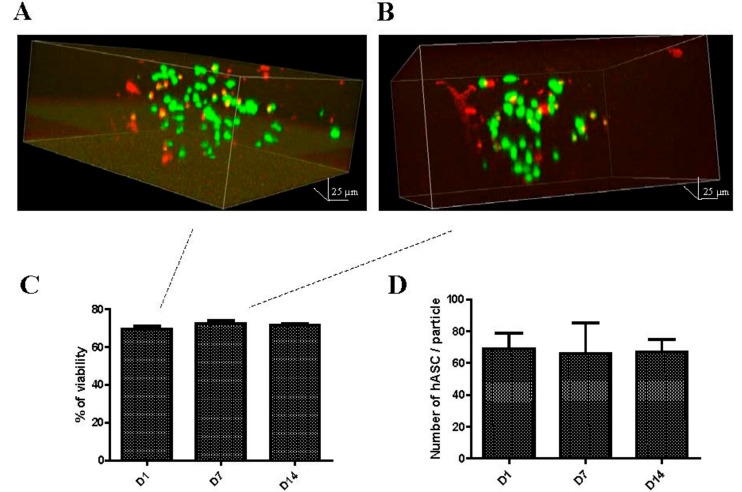
Human adipose-derived stromal cells (hASCs) viability after encapsulation in a Si-HPMC particle. Viable (green) and dead (red) cells were imaged using confocal microscopy and a Live/Dead assay kit at D1 (**A**) and D7 (**B**). hASCs viability in the Si-HPMC particles was monitored over 14 days of culture using a Live/Dead assay kit and manually determined using ImageJ^®^ software. (**C**) Determination of the number of cells per particle was performed after using the Live/Dead assay kit and manually scored using ImageJ^®^ software (**D**). Scale bar: 25 μm.

The viability of the encapsulated hASCs in Si-HPMC particles was 71 ± 2.9% on average, irrespective of the time after encapsulation (Figure 4C), with the differences in percentage lacking statistical significance. The distribution of the cell viability along the radial axis of the Si-HPMC particles was uniform and no accumulation of dead cells in the center of the particles was detected (Figure 4A,B). In addition, the viable cells did not appear to interact with each other and there was no indication that the cells had clustered.

To determine the number of encapsulated hASCs in the Si-HPMC particles, several CSLM images with steps of 10 μm were analyzed using ImageJ^®^ software. The fluorescent cells were manually scored and an average number of 69 ± 10, 66 ± 19, and 67 ± 8 hASCs per Si-HPMC particle was found after 1, 7, and 14 days, respectively, after the encapsulation (Figure 4D). These results were not statistically different.

## 3. Discussion

Cell encapsulation in biomaterials facilitates the injection of MSCs and decreases both the extensive cell death that tends to occur upon injection and the capacity of the MSCs to migrate [15,45]. In this study, MSCs were isolated from human adipose tissue, which contains relatively large numbers (5%) of MSCs [10]. For potential applications in biomedical engineering, it is essential to generate particles with optimal injection properties for in vivo studies: sphericity, reproducibility, and size monodispersity.

Therefore, this study sought to generate reproducible Si-HPMC hydrogel particles by a droplet-based millifluidics method. The aims of this work were to (i) verify the compatibility of Si-HPMC hydrogel rheological properties with millifluidic techniques, (ii) determine the feasibility of generating monodispersed and reproducible particles from a Si-HPMC hydrogel using a novel and original droplet-based millifluidics method, and (iii) assess the ability of this technique to support the in vitro viability of encapsulated hASCs in Si-HPMC particles.

Si-HPMC is a semi-synthetic polymer that undergoes condensation and crosslinking when the pH decreases by the addition of an acidic *N*-(2-Hydroxyethyl)piperazine-*N*′-(2-ethanesulfonic acid)(HEPES) buffer [46]. This step needs to be undertaken with great care as numerous air bubbles can become embedded inside Si-HPMC hydrogels during the mixing step using Luer-Lock syringes containing Si-HPMC solution and HEPES buffer to initiate the decrease of pH and therefore the Si-HPMC crosslinking. Moreover, a slight chemical modification of the glycidoxypropyltrimethoxysilane (GPTMS) grafting rate on HPMC (0.6% *w*/*w* of silane) can lead to pronounced changes in the macroscopic behavior [24].

Our rheological studies confirmed previous results that demonstrate that this hydrogel has characteristics of a shear-thinning fluid, with a decrease in the viscosity observed as the shear rate increases [24]. Shear-thinning fluids are better integrated in capillary flow methods as no flow velocity fluctuations occur during the flow within the tubing. In 2005, Vinatier et al. showed that Si-HPMC hydrogel became a solid hydrogel 30 min after the initiation of crosslinking and that complete crosslinking occurred after 12 days with a maximum *G*′ of 190 Pa [46], which is in keeping with the results of our study. Regarding Si-HPMC crosslinking, increasing temperature reduced the gelation time by a factor of ~4, which is qualitatively in keeping with the findings reported by Fatimi et al. [47]. These authors also found that there was a linear relationship between Ln (*t_gel_*) and 1/T applied with an activation energy of the condensation reaction E_a_ = 74.3 kJ·mol^−1^. The decrease in the gelation time with the increase in temperature at a fixed pH could be explained by the catalytic action of temperature on the silanol condensation [48]. Crosslinking of the Si-HPMC was therefore carried out at room temperature in order to reduce the rate of the chemical condensation reaction and to avoid curing in the millifluidic device. The particles were then collected in complete medium at 37 °C in order to ensure better cell survival and to complete the crosslinking of the Si-HPMC chains. The choice of the cell encapsulation method appears to be suitable for Si-HPMC as a result of its physicochemical properties and crosslinking mechanism.

The advantages of the droplet-based millifluidics method are that it requires a small amount of engaged volumes and that it generates particles with a uniform spherical shape and monodispersed size. Our results show that this encapsulation method supported hASCs survival and that it is suitable for hydrophilic biomaterials such as Si-HPMC. The difficulty in generating reproducible Si-HPMC particles using droplet-based millifluidics lies not only in finding the optimal dispersed and continuous flow rates, but also in the optimization of crosslinking off-line in the collection bath in order to avoid the coalescence of the particles. An appropriate stirring rate using a stirring paddle and a controlled temperature allowed for the production of uniform and spherical particles. In this study, the discrepancies observed in the sizes determined by light microscopy and laser diffraction could be due to the heterogeneous swelling of the Si-HPMC particles, depending on the solvent used (PBS vs. water). Tuning dispersed and continuous flow rates in conjunction with the variation of the internal diameter of the capillary tubing was successfully applied by Martins et al. to generate alginate capsules with diameters ranging from 140 μm to 1.4 mm, according to the variation of flow rates [37]. These results led to the conclusion that droplet-based millifluidics is a versatile and easy-to-use method for the production of a broad range of microparticles of different sizes for encapsulation purposes. In addition, the use of a T-junction configuration for the droplet production allowed for more than 500 particles to be generated per hour.

In order to facilitate the development of hydrogel-assisted hASCs therapies, the particles size can be modulated according to their application. The particles size is governed by a compromise between three criteria: (i) the number of cells that need to be injected in order to achieve the desired therapeutic effect, (ii) the site of the injection, and (iii) the quality of the exchange between the particles and their external environment. In this study, droplet-based millifluidics allowed the generation of 200-μm Si-HPMC monodispersed particles. This is compatible with potential human articular injection for inflammatory disease. The application of a *Q_d_/Q_c_* ratio of 0.1 in droplet-based millifluidics was hence a good compromise for the production of monodispersed Si-HPMC particles with this size, proving to be compatible with this objective. This size is controllable and can be modified by the variation of the flow rates of the different phases for numerous animal models [41]. Particle shape is also a parameter that has an impact on the injectability and the biocompatibility of hydrogels. Indeed, it has been reported that a non-spherical particle shape did not promote their injectability and, more importantly, induced in vivo inflammation [49,50]. The volume of each spherical particle was therefore of 4.2 nL, calculated based on a particle radius of 100 μm in PBS, allowing the in vivo injection of several thousand Si-HPMC particles loaded with hASCs.

The present study also relies on the diffusion of macromolecules of different sizes into the particles. It has been shown that diffusion is affected by the mechanical stress applied on the particles in vivo, which depends greatly on the elasticity, the degree of swelling, and the charge density of the hydrogel. These parameters and their interactions create a complex environment that determines the diffusion and duration kinetics [51]. In the present study, FITC-dextrans (*M_w_* 20, 250, and 2000 kDa) were selected due to their extensive application and ease of use in diffusion studies [52]. These results were analyzed in terms of the hydrodynamic size (instead of the molecular weight) of the dextrans (i.e., branched polysaccharides). The relation between the molecular weight and the hydrodynamic radius of branched polysaccharides is represented by the following expression from Wyatt Technologies (data not shown):

(1) $$M_{w}={\left[1.4782\times R_{h}\right]}^{\left(1.8136\right)}$$

where *M_w_* is the molecular weight (kDa) and *R_h_* is the hydrodynamic radius (nm). Using this equation, the 20, 250, and 2000 kDa fluorescently labeled dextrans were determined to have hydrodynamic diameters of 7, 28.4, and 89.4 nm, respectively.

Given that the average mesh size of the 2% Si-HPMC hydrogels was 32 nm, it would be reasonable to assume that the 20 and 250 kDa fluorescently labeled dextrans should be able to freely diffuse into the Si-HPMC particles while the 2000 kDa dextran should be maintained outside the particles due to steric hindrance. The results shown in Figure 4 indicate that this hypothesis is at least partially true, although the kinetics of the permeability of the 20 and 250 kDa FITC-dextrans appear to be very slow. This slow diffusion process can also arise from heterogeneity in the pore size at the surface of the Si-HPMC particles. The average mesh size compatible with the free diffusion of macromolecules with sizes less than 32.1 nm would, however, not reflect the discrepancies that could exist between the mesh size inside the hydrogel and the pore size in proximity to the surface of the hydrogel. Recently, the diffusion of FITC-dextrans in 1-mm Si-HPMC particles, obtained using a dispersion-emulsification process, revealed that the 20 and 250 kDa FITC-dextrans diffused faster and that, after 150 min of incubation with the Si-HPMC particles, the fluorescence intensity of the 20 kDa FITC-dextran reached equilibrium (i.e., ratio = 1), while the fluorescence intensity ratio was 0.7 for the 250 kDa FITC-dextran. This faster diffusion was therefore attributed to the larger size of the Si-HPMC particles. In addition, no fluorescence intensity was detected with the 2000 kDa FITC-dextran, which is in accordance with the present study [29]. The 20 kDa FITC-dextran was of particular interest, as the therapeutic factors secreted by stimulated hASCs have molecular weights that range from about 10 to about 45 kDa [53,54]. A thorough study using environmental microscopy could be very useful to probe the internal and external structures of Si-HPMC particles. These particles would, however, be adapted to allow the diffusion of essential nutrients for the viability of encapsulated hASCs, as well as the diffusion of therapeutic factors secreted in an inflammatory environment by encapsulated hASCs.

As hASCs are thought to mainly exert their therapeutic potential by the secretion of immunomodulatory, pro-angiogenic, anti-apoptotic, anti-fibrotic, and anti-inflammatory factors, encapsulated hASCs must remain viable and the average number of cells per particle has to be sufficient [55]. In the present study, the viability of encapsulated hASCs in Si-HPMC particles was estimated to be 70% after 14 days. As the encapsulation technique involves some degree of mechanical shear, it probably results in a slight reduction of cell viability. Unlike the direct injection of cells into the body with a conventional injection system, millifluidics can substantially reduce the extent of cell death. This result confirms that Si-HPMC particles obtained by millifluidics support the diffusion of nutrients, vitamins, and glucose essential for the survival of the encapsulated hASCs. In 2018, Figueiredo et al. showed that the diffusion of glucose through Si-HPMC hydrogels was correlated directly with the average distance between the polymer nodes in the hydrogel network, while the diffusion of oxygen was found to be the limiting factor for cell viability in Si-HPMC hydrogels [56]. In addition, the present study did not find that the dead cells were specifically localized at the center of the particles. Thus, it would appear that a sufficient level of nutrients, glucose, and oxygen reached the center of the particles by diffusion. This strongly suggests that, due to the suitable extent of diffusion obtained, the particle size did not appear to constitute a limiting factor for cell viability in this range of particle sizes. At 24 h after the encapsulation, the average number of live hASCs per Si-HPMC particle was estimated to be approximately 70. This average cell number remained constant for two weeks, suggesting that the cells in the Si-HPMC particles did not proliferate. This result is in line with the lack of hASC adhesion when encapsulated in a Si-HPMC hydrogel, as demonstrated by Moussa et al. [57]. With a negatively charged cytoplasm membrane, hASCs adhesion depends on the charge of the hydrogel matrix. The overall neutral behavior of the Si-HPMC polymer does not provide a favorable environment for the adhesion and proliferation of the encapsulated hASCs. For most cell therapy applications, the apparent inability of the encapsulated hASCs to proliferate is, however, not a drawback. Therefore, the findings of the present study are a further indication that cellular microenvironments can be developed that permit the release of soluble therapeutic factors after the injection of cells, while also preventing the triggering of inflammation in degenerative diseases such as osteoarthritis.

## 4. Materials and Methods

### 4.1. Materials

Hydroxypropylmethylcellulose (HPMC) (Methocel^TM^E4M) was purchased from Colorcon-Dow chemical (Bougival, France). Glycidoxypropyltrimethoxysilane (GPTMS) was obtained from Acros (Geel, Belgium). Hank’s Balanced Sodium Salt (HBSS), Dulbecco’s Modified Eagle Medium high glucose (4.5 g/L) (DMEM), phosphate buffered salt (PBS) without calcium chloride and magnesium chloride, penicillin/streptomycin, and trypsin/EDTA (0.05%/0.53 mM) were obtained from Invitrogen (Paisley, UK). 4-(2-hydroxyethyl)-1-piperazineethanesulfonic acid (HEPES), olive oil, fluorescein isothiocyanate (FITC)-dextrans, collagenase crude type I A, and trypan blue were obtained from Sigma-Aldrich (St. Louis, MO, USA). Fetal calf serum (FCS) was purchased from Dominique Dutscher (Brumath, France). The Live/Dead assay kit was obtained from Molecular Probes (Leiden, The Netherlands). Twelve-well plates (ref. 3512) were purchased from Corning (Boulogne Billancourt, France).

PolyEtherEtherKetone (PEEK) tubing for millifluidics were purchased from CIL, Cluzeau Info Labo (Sainte Foy la Grande, France).

### 4.2. Synthesis of the Hydrogel

The synthesis of the silanized-HPMC (Si-HPMC) was performed by grafting 14.24% (*w*/*w*) GPTMS onto HPMC in a heterogeneous medium as previously described [23]. Lyophilized Si-HPMC powder was solubilized (4% *w*/*v*) in 0.1 M NaOH under constant stirring for 24 h. The solution was then sterilized by steam autoclave (121 °C, 20 min). A Si-HPMC solution can be made to undergo crosslinking by a decrease in the pH. The sterilized solution was therefore mixed with one volume of HEPES buffer (*v*/*v*) (pH 3.55), in order to initiate the formation of a crosslinking of Si-HPMC chains at a final concentration of 2%.

### 4.3. Rheological Characterization of the Si-HPMC Hydrogel

#### 4.3.1. Characterization of the Si-HPMC Polymeric Solution

Steady shear measurements were carried out to determine the viscosity of the unsterilized 4% Si-HPMC solution according to the simplified Cross equation [58].

(2) $$\eta =\frac{\eta_{0}}{1+{\left(\lambda {\dot{\gamma }}_{c}\right)}^{n}}$$

where η_0_ is the limiting Newtonian viscosity at a low shear rate (Pa.s), λ is the relaxation time (the inverse of a critical shear rate ${\dot{\gamma }}_{c}$) (s), and *n* is the exponent of the power law.

Flow measurements were performed at 23 °C, with a fixed shear stress of 1 Pa, using a Rheostress 300 rheometer (ThermoHaake^®^, Karlsruhe, Germany) equipped with a titanium cone-plate geometry (60 mm in diameter, 1° cone angle). The gap between the truncation and the plate was 0.052 mm.

#### 4.3.2. Characterization of the Si-HPMC Hydrogel

To study the gel times of 2% Si-HPMC, dynamic frequency experiments were carried out using a Rheostress 300 rheometer (ThermoHaake^®^, Karlsruhe, Germany) equipped with a titanium cone-plate geometry (60 mm in diameter, 1° cone angle), immediately after the initiation of the Si-HPMC crosslinking. The storage (*G*′) and loss (*G*′′) moduli were determined as a function of time at five oscillation frequencies (0.30 Hz, 0.50 Hz, 1 Hz, 1.80 Hz, and 3.2 Hz). This assessment was operated under a stress amplitude of 1 Pa at 23 or 37 °C. The temperature was controlled by an external thermal bath. The gelation time (*t_gel_*) was calculated as the time at which *tan δ*(= *G*′′/*G*′) becomes independent of the frequency, in accordance with the criterion defined and proposed by Winter and Chambon [43,44].

To estimate the Si-HPMC hydrogel mesh size ξ (i.e., the average distance between the polymer entanglements in the hydrogel network), the Si-HPMC solution was crosslinked in a 12-well plate for 24 h at 37 °C. Hydrogel with a height of 5 mm and a diameter of 22 mm was then removed from the 12-well plate. Dynamic shear stress sweep measurements, with stress amplitudes ranging from 0.1 to 1000 Pa and a fixed frequency of 1 Hz, were carried out at 23 °C using a Mars rheometer (ThermoHaake^®^, Karlsruhe, Germany) equipped with a plate-plate geometry (20 mm in diameter). The Si-HPMC hydrogel mesh size ξ (m) was determined according to the Flory equation:

(3) $$\xi ={\left[\frac{k_{B}T}{G^{\prime }}\right]}^{\left(\frac{1}{3}\right)}$$

with *k_B_* representing the Boltzmann constant (J/K) and *T* the temperature (K) [59]. The average storage modulus (*G*′) (Pa) in the linear regime on triplicate samples was determined to calculate the mesh size of the hydrogel.

### 4.4. Preparation of Si-HPMC Particles Using Millifluidics

**Figure 5:**
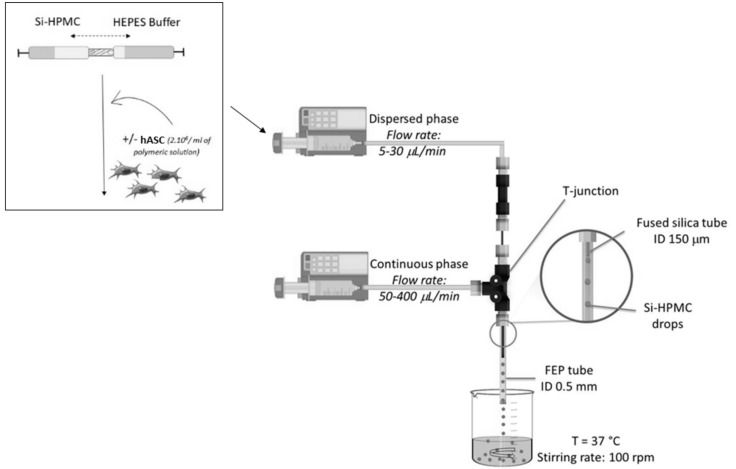
The droplet-based millifluidics device used to produce Si-HPMC particles. The control of temperature and stirring rate off-line in the collection bath were critical parameters that were optimized to avoid the coalescence of the particles and to maintain cell viability. The dispersed phase was comprised of Si-HPMC solution in the presence of freshly prepared HEPES buffer +/− loaded hASCs.

A millifluidics device with a T-junction configuration was used to produce Si-HPMC particles (Figure 5) [60]. The dispersed phase, the mixing of the Si-HPMC solution with freshly prepared HEPES buffer, was pumped (Pilote C, Fresenius Kabi^®^, France) through a fused silica capillary tube (interior diameter (ID) = 150 μm and outside diameter (OD) = 375 μm) at a rate that varied between 5 and 30 μL/min. The continuous phase, olive oil compatible with biomedical applications, was pumped through a Teflon tube (ID = 0.5 mm and OD = 1.571 mm) at a rate that varied between 50 and 400 μL/min. The Si-HPMC hydrogel and the oil co-flowed in the Teflon tube (ID = 0.5 mm, OD = 1.571 mm, and length = 10 cm). The Si-HPMC drops were formed and dispersed in the continuous oil phase. The crosslinking of Si-HPMC, starting in the drops due to the decrease of pH after the addition of HEPES buffer, continued in the collection bath. A controlled stirring rate of 100 rpm using a rotating paddle and a temperature of 37 °C in the collection bath was shown to be crucial to avoid the coalescence of the Si-HPMC particles and to maintain the viability of the cells. After stirring for 3 h, the particles were sieved using a 100-μm mesh filter unit and rinsed using complete medium (DMEM containing 1% penicillin/streptomycin and 10% FCS). The Si-HPMC particles were then incubated at 37 °C in PBS until use.

### 4.5. Characterization of the Si-HPMC Particles

#### 4.5.1. Shape and Size

Particles were observed by light microscopy (Leica microsystems CMS GmbH, Type 11 090 137 002, Leica Biosystems, Nussloch, Germany) to investigate their shape, while the particle size was measured using a Mastersizer 3000 Laser (Malvern Instruments, Malvern, UK).

#### 4.5.2. Diffusion Properties of the Si-HPMC Particles

The diffusion properties were studied by immobilizing Si-HPMC particles at the bottom of Lab-Tek chambers and followed by their incubation in 1 mg/mL solutions of fluorescently labeled dextran (molecular weights (*M_w_*) of 20, 250, or 2000 kDa), for 18 h at room temperature. After incubation (6 h, 12 h, and 18 h), the particles were observed by confocal laser scanning microscopy (CLSM) (Nikon A1R Si, Champigny sur Marne, France; excitation wavelength of 488 nm, emission wavelength of 520 nm) in order to quantify the amount of fluorescently labeled dextran that had diffused into the Si-HPMC particles. The images were analyzed with NIS-Elements software to determine the fluorescence intensities inside the particles and outside (i.e., in the FITC-dextran solution). The ratio between the internal and the external fluorescence was then calculated. A ratio of 1 indicates that the fluorescence intensity was identical inside and outside the particles and that equilibrium was reached. The results were expressed as the internal/external fluorescence ratio over time.

### 4.6. Isolation and Culture of the hASCs

Human adipose-derived mesenchymal stem cells (hASCs) were isolated from subcutaneous adipose tissue of patients undergoing liposuction [61]. All of the protocols were approved by the biomedicine agency. ASCs were obtained from human patients undergoing liposuction and who had given written consent (Agence de BioMédecine n° PFS08-018, legislation codes L.1211-3 to L.1211-9, approval date: 9 September 2008). Briefly, lipoaspirate was washed five times in HBSS and then digested for one hour at 37 °C under constant stirring, in a solution of 0.025% collagenase in HBSS. The collagenase treatment was inactivated by the addition of an equal volume of complete medium. After 5 min of centrifugation (260× *g*, 4 °C), the lower phase containing the stromal vascular fraction was collected, homogenized, filtered through a 70-μm cell strainer, and centrifuged for 8 min (260× *g*, 4 °C). The cells were suspended in complete medium, seeded at 5000 cells/cm^2^, and incubated at 37 °C in a humidified atmosphere containing 5% CO_2_. After 2–3 days of incubation, the non-adherent cells were removed by successive washes.

### 4.7. hASCs Encapsulation

The cells were used at passage 5 (population doubling level (PDL) of 13.6). After being harvested using a trypsin/EDTA solution, the hASCs were counted and then loaded into Si-HPMC hydrogel. After the induction of crosslinking by the addition of HEPES buffer, 2.10^6^ hASC (150 μL) was suspended in 1 mL of a sterile Si-HPMC hydrogel [46]. After droplets were generated in the millifluidics device, the hASCs loaded in the Si-HPMC particles were placed in complete medium (DMEM containing 1% penicillin/streptomycin and 10% FCS) for 3 h with stirring at 100 rpm and 37 °C. The Si-HPMC particles were then collected and incubated in complete medium, after the removal of the oily continuous phase.

### 4.8. hASCs Viability

The cells encapsulated in Si-HPMC were cultured in complete medium for up to 14 days at 37 °C in a humidified atmosphere containing 5% CO_2_. The medium was changed every 2 days. After encapsulation, the hASCs viability in the Si-HPMC particles was followed from 24 h to 14 days of culture using a Live/Dead assay kit. The Si-HPMC particles were recovered, washed in PBS, and incubated for 45 min in the combined Live/Dead assay reagents. The labeled cells were imaged by confocal microscopy using an inverted fluorescence microscope (Nikon Eclipse TE 200 E, Badhoevedorp, The Netherlands). ImageJ^®^ Software (version 1.8.0, NIH, Bethesda, MD, USA) was used to perform hASC viability calculations (the ratio of the number of live cells and the total number cells).

The average number of encapsulated hASCs per particle was manually assessed by confocal laser scanning microscopy (CLSM) (Nikon A1R Si, Champigny sur Marne, France) after analyses of approximately 500 images sections (section thickness of 10 μm) using ImageJ^®^ Software.

### 4.9. Statistical Analysis

All of the experiments were performed with replicate samples from independent conditions (*n* = 3). Results of a representative experiment are presented as the mean of three independent replicates, and the error bars represent the standard error of the mean. The comparative studies of means were performed with GraphPad^®^ software by using one-way ANOVA followed by a post hoc test with a statistical significance of *p* < 0.05.

## 5. Conclusions

In light of the considerable amount of encouraging data in the literature, ASCs encapsulation has a promising future in the treatment of inflammatory diseases. However, hASCs encapsulation remains rarely used for cell therapies. In this context, a novel and original approach based on droplet-based millifluidics was developed for the encapsulation of hASCs in injectable and biocompatible particles produced from Si-HPMC hydrogel. This versatile and effective tool allowed the generation of particles that fulfill three essential requirements for further in vivo study: sphericity, reproducibility, and size monodispersity. Spherical particles of 200 μm in diameter with an excellent reproducibility were obtained, which is a size that is suitable for injection in a large animal model. In addition, the successful encapsulation of hASCs in Si-HPMC particles was achieved and the cells remained viable for 14 days in vitro. The Si-HPMC particles’ diffusion properties suggest that the diffusion of low-molecular-weight FITC-dextran was compatible with the diffusion of nutrients essential for the survival of the hASCs after their encapsulation. Droplet-based millifluidics therefore appears to be a promising non-cytotoxic method for the encapsulation of hASCs. Further developments are needed, however, in order to ensure better encapsulation performance, particularly by adding a third channel in the millifluidic setup, while ensuring cell sterility and the reduction of the “cellular stress” phenomenon.
